# Plasma-based Raman spectroscopy for early detection of acute myocardial infarction in murine models

**DOI:** 10.1038/s41598-025-30292-y

**Published:** 2025-12-10

**Authors:** Chengyou Jia, Chunyan Duan, Jing Sun, Shijun Chen, Zhengshi Wang, Lin Sun, Xiaoli Yang, Xiankai Li, Zhongwei Lv

**Affiliations:** 1 Department of Nuclear Medicine, Clinical Nuclear Medicine Center, Imaging Clinical Medical Center, Institute of Nuclear Medicine, Institute of Clinical Mass Spectrometry Applied Research Center, School of Medicine, Shanghai Tenth People’s Hospital, Tongji University, Shanghai, 200072 China; 2 Department of Cardiovascular medicine, Shanghai Tenth People’s Hospital, Tongji University School of Medicine, Shanghai, 200072 China; 3 Department of Mechanical Engineering, School of Mechanical Engineering, Tongji University, 4800 Cao’an Highway, Shanghai, 201804 China; 4 Department of Thyroid and Brest, School of Medicine, Shanghai Tenth People’s Hospital, Tongji University, Shanghai, 200072 China; 5 Molecular Imaging Center, Shanghai Institute of Materia Medica, Chinese Academy of Sciences, Shanghai, 201203 China; 6 Shanghai Public Health Clinical Center, Fudan University, Shanghai, 200003 China

**Keywords:** Acute myocardial infarction, Raman spectroscopy, Machine learning, Plasma, Biological techniques, Biomarkers, Cardiology

## Abstract

Acute myocardial infarction (AMI) is a leading cause of cardiovascular mortality. Current diagnostic dilemma suffers from limited sensitivity, insufficiently timely and effective specificity. To address this dilemma, Raman spectroscopy, a rapid and non-invasive technique with significant potential for plasma metabolic profiling, although AMI involves rapid metabolic alterations, diagnostic approaches based on plasma metabolites remain underexplored. In murine AMI model induced by coronary artery ligation, we acquired Raman spectra from ultrafiltered plasma samples 8 h post-surgery. We identified eight distinct Raman peaks, assigned to amino acids, lipids, and nucleic acids, which collectively differentiated AMI group (*n* = 27) from the Sham group (*n* = 27). To ensure optimal accuracy, we employed five different algorithms including SVM, LR, RF, LDA and PLS-DA to analyze the Raman spectra, both RF and LDA achieved the highest accuracy of 79.6%, specificity of 85.2%, and sensitivity of 74.1%. Furthermore, metabolomic analysis revealed significant down-regulation of most lipid classes, aligning with the downregulation observed in the Raman peaks at 2885 cm⁻¹ and 2940 cm⁻¹. These results demonstrate a high concordance between plasma metabolic profiling via Raman spectroscopy and MS analysis. The integration of Raman spectroscopy with machine learning has remarkable potential for the early and accurate diagnosis of AMI, offering a promising approach for clinical translation.

**Supplementary Information:**

The online version contains supplementary material available at 10.1038/s41598-025-30292-y.

## Introduction

Acute myocardial infarction (AMI), characterized by irreversible cardiomyocyte necrosis due to prolonged coronary ischemia, remains a leading global cause of cardiovascular mortality^1,2^. Current diagnosis of AMI generally relies on electrocardiogram (ECG) findings and cardiac biomarkers such as troponin and creatine kinase^3–5^. However, ECG exhibits critical limitations: while it achieves > 90% sensitivity in ST-segment elevation myocardial infarction (STEMI), its diagnostic accuracy plummets to 57% in non-ST-segment elevation myocardial infarction (NSTEMI) NSTEMI constitutes 60% of AMI cases^6,7^. Although high-sensitivity cardiac troponin (hs-cTn) assays have improved detection thresholds, their accuracy may be affected by kidney impairment or chronic myocardial injury^8–11^. Consequently, there is an urgent need for novel biomarkers and rapid diagnostic platforms for the early diagnosis of AMI.

Metabolomics has been extensively utilized for cardiovascular diagnostics, capturing dynamic pathophysiological changes through comprehensive small-molecule analysis^12,13^. Mass spectrometry (MS), which is regarded as the gold standard for metabolomic analysis, facilitates the accurate quantification of low-abundance metabolites and is instrumental biomarkers discovery. Elevated levels of L-homocysteine sulfinic acid, cysteic acid, and carnitine have been identified as potential biomarkers for the early detection of AMI^14^. Additionally, the proportions of short-chain and long-chain fatty acids have demonstrated diagnostic significance for AMI in younger populations^15^. A diagnostic model comprising five metabolites, including L-aspartic acid, arachidonic acid, palmitoleic acid, D-aspartic acid, and palmitelaidic acid, has been proposed as a biomarker set for the early diagnosis of NSTEMI patients^16^. However, MS suffers from high operational costs, time-consuming procedures, and intricate sample preparation, which limit its effectiveness in the early diagnosis of AMI.

Raman spectroscopy, an optical technique based on the inelastic scattering of light, offers intricate chemical fingerprints based on molecular vibration. This method is characterized by its cost-effectiveness, non-invasive nature, and high sensitivity, enabling the rapid analysis of small sample volumes^17^. Furthermore, Raman spectroscopy can be integrated with artificial intelligence algorithms to develop diagnostic classification models for automated identification^18,19^. Raman spectroscopy has demonstrated remarkable success in cancer diagnostics^20–22^, yet its potential in AMI-related metabolic profiling remains underexplored.

**Fig. 1:**
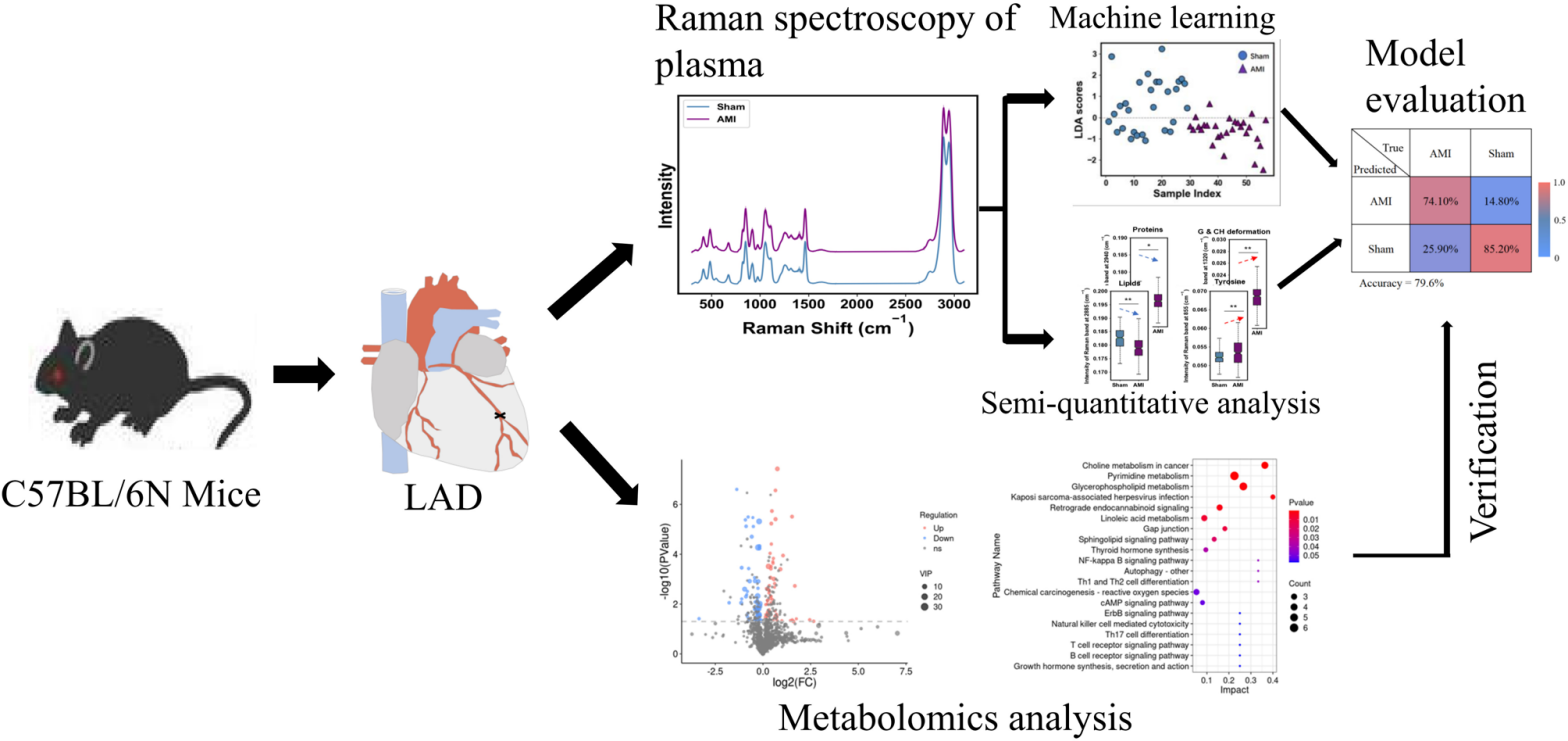
This schematic illustrates an innovative workflow for diagnosing AMI using Raman spectroscopy integrated with machine learning. Initially, AMI was modeled in mice via coronary artery ligation, followed by collection of plasma samples 8 h post-surgery for Raman spectral and MS analysis. The study identified eight characteristic Raman peaks—corresponding to amino acids, lipids, and nucleic acids—that effectively distinguished the AMI group from the sham group. Five machine learning algorithms were applied to process the spectral data, with the optimal model achieving an accuracy of 79.6% by RF and LDA. Metabolomic validation confirmed that downregulated lipid metabolism correlated consistently with changes in the Raman spectral features.

Herein, we developed a Raman-AI integrated strategy for AMI detection. Using a murine AMI model induced by coronary ligation, we acquired plasma Raman spectra at 8 h post-ischemia. The integration of five machine learning algorithms with Raman spectral data enabled discrimination between the two groups and identification of significant metabolites associated with AMI. Plasma metabolomics analysis validated the reliability of Raman spectroscopy in metabolite identification. Our findings suggest that Raman-based metabolic profiling holds promise for the early and accurate diagnosis of AMI. A graphical workflow of entire study is presented in Fig. 1.

## Materials and methods

### Materials and chemicals

Acetonitrile (Lot: 100030), ammonium acetate (Lot: 73594), methanol (Lot: 900688), and ammonia (Lot: 54380) were procured from Merck, with all reagents exhibiting LC-MS grade purity. Ultrapure water was generated utilizing a Milli-Q water purification system (Merck). Pentobarbital sodium was obtained from Sinopharm Chemical Reagent Co., Ltd. (Shanghai, China). Additionally, commercially available Mouse cTn I assay kits (JK-E3986, Shanghai Jingkang Biotechnology. Co. Ltd.

### Animals and the AMI model

All animal experimentation protocols received approval from the Laboratory Animal Management Committee of Shanghai Tenth People’s Hospital (SHDSYY-2023-4983), and were in accordance with the relevant guidelines and regulations. C57BL/6 wild-type mice, aged 8 weeks and weighing approximately 20 ± 2 g, were sourced from N Shanghai Jihui Laboratory Animal Co., Ltd. (Shanghai, China). The mice were maintained in a controlled environment with a 12-hour light/dark cycle, a constant temperature of 23 ± 2 °C, and a relative humidity of 50%. Following a 7-day acclimatization period, the mice were randomly assigned to two groups: Sham and AMI. Anesthesia was induced via intraperitoneal injection of 1% sodium pentobarbital. The AMI model in mice was established through the permanent ligation of the left anterior descending artery (LAD), following the methodology described by Gao et al. (2010)^23^. Sham surgery was conducted using the same procedural steps, with the exception of LAD ligation. The murine models across all experimental groups underwent immediate evaluation via electrocardiogram following surgical intervention. The blood samples were collected in ethylene diamine tetraacetic acid (EDTA) anticoagulant tubes post-surgery after an 8-hour interval and were centrifuged at 3000 rpm for 10 min to isolate plasma samples, which were then stored at -80 °C. The mice were euthanized through cranio-cervical dislocation. The plasma levels of Cardiac Troponin I (cTn I) were quantified using commercially available Mouse cTn I assay kits (JK-E3986, Shanghai Jingkang Biotechnology. Co. Ltd.). Morphological alterations in cardiomyocytes were assessed through Hematoxylin-Eosin (HE) staining. The cardiac tissues from the mice were preserved in 4% buffered formalin for a duration of 24 h, subsequently fixed, embedded in paraffin, and sectioned into 5 μm slices for HE staining analysis. The study followed ARRIVE guidelines (http://www.nc3rs.org.uk/arrive-guidelines).

### Raman spectroscopy

To reduce the interference from high-abundance proteins and currently applied serum AMI markers, 40 µL of plasma ultrafiltrate was prepared by ultracentrifugation using a 10 kD molecular weight cutoff (MWCO) filter (UFC501024, Thermo Scientific™, USA)^24^. For each sample, a single 3 µL droplet of plasma ultrafiltrate was placed on calcium fluoride slides and allowed to partially dry at room temperature (22–26 °C). Raman spectroscopy detection was Under a clear and uniform microscopic field of view, Raman spectra of the filtered plasma samples were acquired using the WITec Raman alpha300R confocal Raman imaging system, we performed 3 × 4 array comprising 12 points within at least 40 μm×60 μm area of air-dried plasma droplet. A 532 nm Nd: YAG laser, operating at an excitation power of 10 mW, was focused onto the sample through a 50 × (0.75 NA, Zeiss) objective, and the resulting Raman signals were detected using a 300 mm spectrograph equipped with a 600 g/mm grating and a charged-coupled device (CCD). Raman spectra were recorded in the range of 0 to 3600 cm^− 1^ with an integration time of 5 s per spectrum. To mitigate instrumental discrepancies between the Raman equipment and the sensing chip, the spectrometer was calibrated using a silicon crystal (520.6 cm^− 1^ peak). A total of twelve Raman spectral measurements were collected from each plasma droplet. The spectra were interpolated within the fingerprint region spanning 400 to 3200 cm^− 1^. Original Raman spectra were collected and processed using standard procedures including background subtraction, cosmic ray removal, baseline calibration, and normalization (scaling to a maximum peak intensity of for all spectra with the same condition. These steps were executed using the WITec Project SIX software (Wec™ 300R, Ulm, Germany).

### Machine learning

Normalized the Raman spectra are averaged and then utilized the for construction of models that distinguish the Sham and AMI groups. To improve parameter optimization, we employed accuracy scores and Area Under the Curve (AUC) metrics. Five machine learning models used in this analysis including Support Vector Machine (SVM), Logistic Regression (LR), Random Forest (RF), Linear Discriminant Analysis (LDA) and Partial Least Squares Discriminant Analysis (PLS-DA).

### Metabolomics methodology

Non-targeted metabolomics analysis was carried out by Applied Protein Technology company Co.,Ltd (Shanghai, China). (https://www.aptbiotech.com/) using 150 µL aliquot of the thawed plasma. Briefly, precooled methanol/acetonitrile/water solution in a 2:2:1 ratio (v/v). Following a 30-second vortex mix and a 30-minute sonication in an ice bath, the samples were incubated overnight at -20 °C to enhance protein precipitation. The samples were then centrifuged at 14,000 g for 20 min at 4 °C and subsequently dried under vacuum. To redissolve the residue, 100 µL of a 1:1 acetonitrile/water solution was added, followed by vortexing and then centrifugation at 14,000 g for 15 min at 4 °C. The resulting sample vials were stored at -20 °C until further analysis.

Chromatographic separation was conducted using a UHPLC system (1290 Infinity LC, Agilent Technologies) equipped with a Waters ACQUITY UPLC BEH Amide column (1.7 μm; 100 × 2.1 mm). The column temperature was maintained at 25 °C, with a flow rate of 0.5 mL/min and an injection volume of 2 µL. The mobile phase A consisted of 25 mM ammonium acetate and 25 mM ammonium hydroxide in water, while mobile phase B was acetonitrile. The linear gradient was programmed as follows: 0–0.5 min, 95% B; 0.5–7 min, 95% to 65% B; 7–8 min, 65% to 40% B; 8–9 min, 40% B; 9–9.1 min, 40% to 95% B; 9.1–12 min, 95% B. Throughout the analysis, samples were maintained at 4 °C in an automatic sampler.

Metabolomic profiling was conducted using an SCIEX Triple TOF 6600 mass spectrometer with information-dependent acquisition (IDA). The electrospray ionization (ESI) source parameters were set as follows: Ion Source Gas1 (Gas1) at 60, Ion Source Gas2 (Gas2) at 60, curtain gas (CUR) at 30, source temperature at 550 °C, and ion spray voltage floating (ISVF) at ± 5500 V. The TOF/MS full scan was performed over a mass range of 60-1000 Da with an accumulation time of 0.20 s, while the TOF-MS/MS full scan was conducted over a mass range of 25-1000 Da with an accumulation time of 0.05 s. In both positive and negative ion modes, the collision energy (CE) was set to 35 ± 15 eV, and the declustering potential (DP) was established at ± 60 V. The mass spectrometer underwent automatic calibration via the calibration delivery system (CDS) after every nine injections.

### Metabolomics data analysis

The raw MS data, which is in wiff.scan. file format, was transformed into MzXML files using the ProteoWizard MS Convert tool before being loaded into the publicly accessible XCMS software. Following sum-normalization, the processed data underwent analysis using the R package ropls, which facilitated multivariate data analysis techniques, including Pareto-scaled principal component analysis (PCA) and orthogonal partial least-squares discriminant analysis (OPLS-DA). To assess the robustness of the model, a 7-fold cross-validation and response permutation testing was employed. The variable importance in projection (VIP) value for each variable within the OPLS-DA model was computed to ascertain its contribution to the classification process. A Student’s t-test was conducted to evaluate the significance of differences between two independent sample groups. Criteria for identifying significantly altered metabolites included VIP > 1 and p-value < 0.05. Ultimately, 64 metabolites in ESI^+^ mode and 44 in ESI^−^ mode were identified as differential metabolites. The Kyoto Encyclopedia of Genes and Genomes (KEGG) pathway enrichment analysis for the altered metabolites was conducted using the KEGG database^25^.

## Results

### AMI mice models construction

**Fig. 2:**
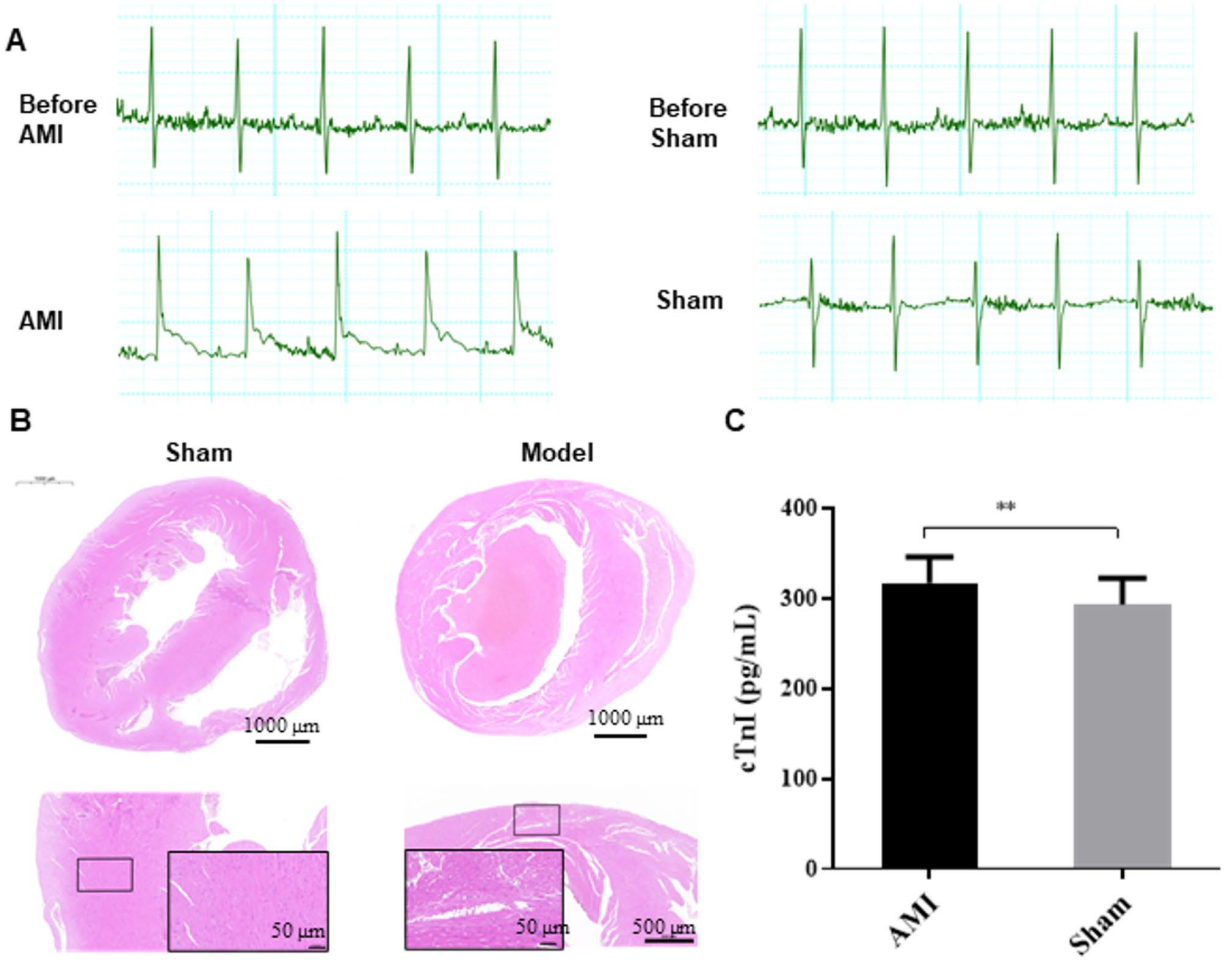
AMI model construction. **(A)** Comparation of ECG assessments between the AMI and Sham groups. **(B)** Hematoxylin-Eosin (H&E) staining of cross-sectional cardiac tissues from murine models in the AMI and Sham groups. **(C)** Plasma cTn I level in the AMI and Sham groups (*n* = 27 each group). ** represents *p* value < 0.01 between AMI vs. Sham group.

C57BL/6 N mice were used to establish a AMI model by permanent ligation of the left anterior descending artery. electrocardiogram (ECG) recordings were obtained before and immediately after surgery to evaluate the success of AMI induction. The AMI group showed significant ST-segment-T wave elevation compared to the Sham group (Fig. 2A). For histological examination, cardiac tissues were fixed, embedded, and subjected to Hematoxylin and Eosin (H&E) staining. Cardiomyocytes in the Sham group maintained a well-organized nuclear architecture. whereas those in AMI group displayed mild disorganization (Fig. 2B), Plasma samples were collected 8 h post-surgery for plasma cTn I assessment, mass spectrometry and Raman spectroscopy analysis. cTn I, a specific plasma biomarker for diagnosing AMI was measured in these samples. The level of plasma cTn I was significantly elevated in the AMI group compared to the Sham group (*p* < 0.01), thereby confirming myocardial injury and infarction (Fig. 2C). Together, these results demonstrate that the AMI model was successful constructed and that 8 h post-surgery is suitable time point for subsequent metabolic and spectral analyses.

### Raman analysis of plasma samples

**Fig. 3:**
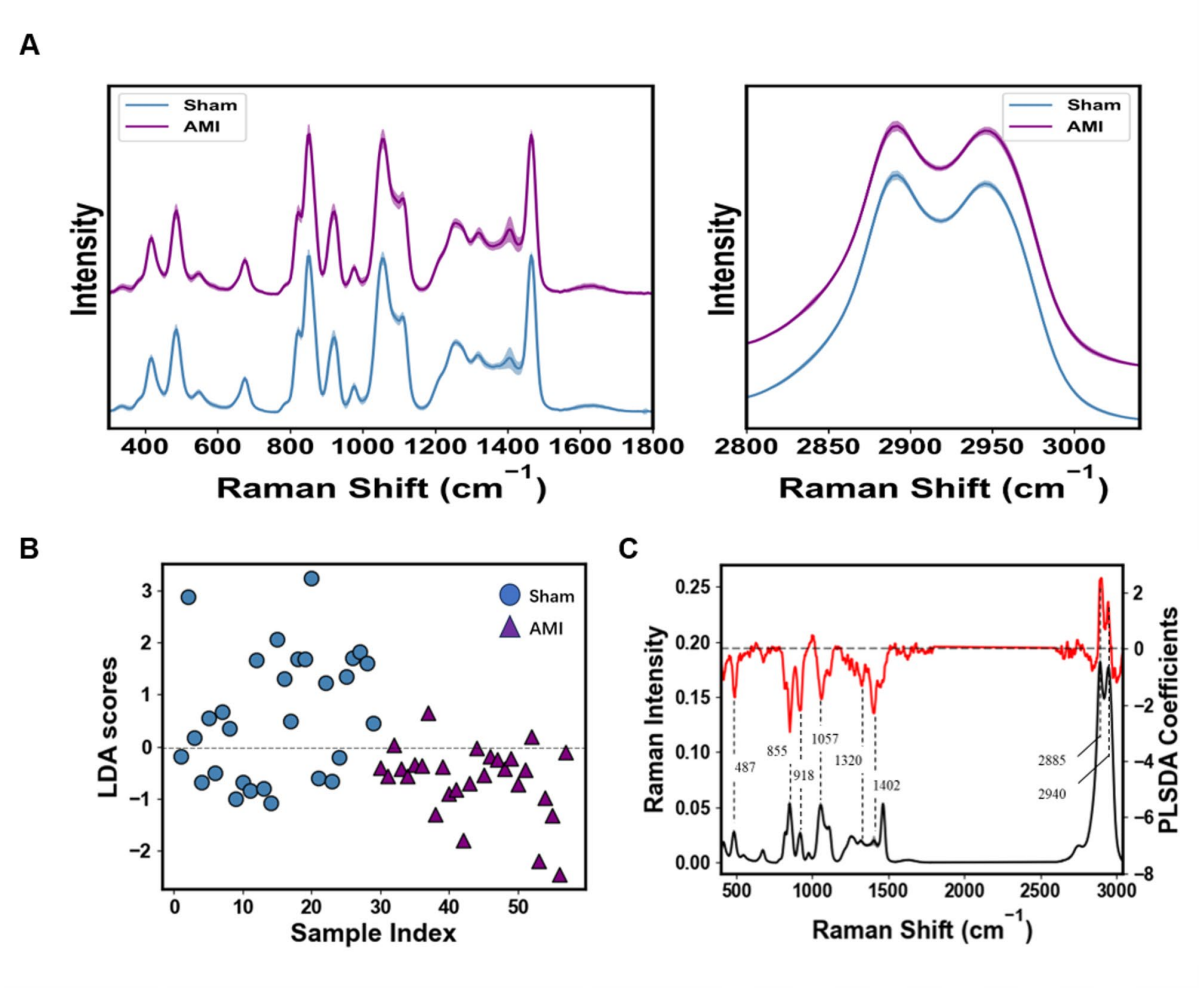
Raman analysis of plasma samples. **(A)** The average Raman spectra of 27 AMI and 27 Sham plasma filtered samples. **(B)** PCA-LDA of AMI vs. Sham showing separation of the two groups. **(C)** Weight coefficient diagram of Raman spectra (red) and averaged Raman spectra (*n* = 638) acquired from 54 mice filtered plasma samples (black).

Raman spectra were acquired from 54 mice plasma filtrate using 532 nm laser with 10 mW power and a 50 × objective lens, detection parameter including accumulation time is 1 s per spectrum and the number of accumulation time is 2. The plasma samples (3 µL) were deposited on CaF_2_ disks, dried for 5 min and then subjected to spectral measurement. To minimize the effects of sample heterogeneity, at least 12 spectra were recorded from each filtered plasma within 40 μm×60 μm area in 3 × 4 array pattern. The average normalized Raman spectra of AMI and Sham group plasma samples are displayed (Fig. 3A). PCA-LDA was applied for data procession, revealing a clear separation between the AMI and Sham groups, with each data point corresponds to an averaged and normalized spectrum from a single plasma sample (Fig. 3B). To further evaluate spectral differences, PLS-DA was applied to model the Raman spectral profiles and classify the samples. The accuracy and AUC of the model varied with 20 factors included in the PLS-DA analysis. The PLS factor is optimized to 3rd factor according to the cross validation, that yielding an accuracy of 0.754 and an AUC of 0.801. Consequently, for all subsequent analyses were conducted using PLS-DA model chosing factors three presented (Supplementary Fig-S1). The weight coefficient derived from the PLS-DA model were visualized to highlight the contribution of individual Raman peaks in distinguishing between AMI and Sham group. A total of eight characteristic Raman peaks were identified at 487 cm^− 1^, 855 cm^− 1^, 918 cm^− 1^, 1057 cm^− 1^, 1320 cm^− 1^, 1402 cm^− 1^, 2885 cm^− 1^ and 2940 cm^− 1^, were identified (Fig. 3C), Their corresponding chemical bonds or molecules summarized in in Table 1.

**Table 1:** Assignments for Raman spectral bands.

Raman spectrum (cm^− 1^)	Assignment	References
487	Glycogen	^26^
855	Proline, tyrosine n(C-C), tyrosine (protein assignment and polysaccharide)	^27^
918	Proline, hydroxyproline	^28^
	Glycogen and lactic acid	^29^
1057	Lipids	^30^
1320	G (DNA/RNA) CH deformation (proteins)	^31^
1402	ʋ (C = O) O^−^ (amino acids aspartic & glutamic acid)	^32^
2885	ʋ_s_ CH_3_, lipids, fatty acids	^32^
2940	C-H c& proteins ʋ_as_ CH_2_, lipids, fatty acids	^33^
		^32^

**Fig. 4:**
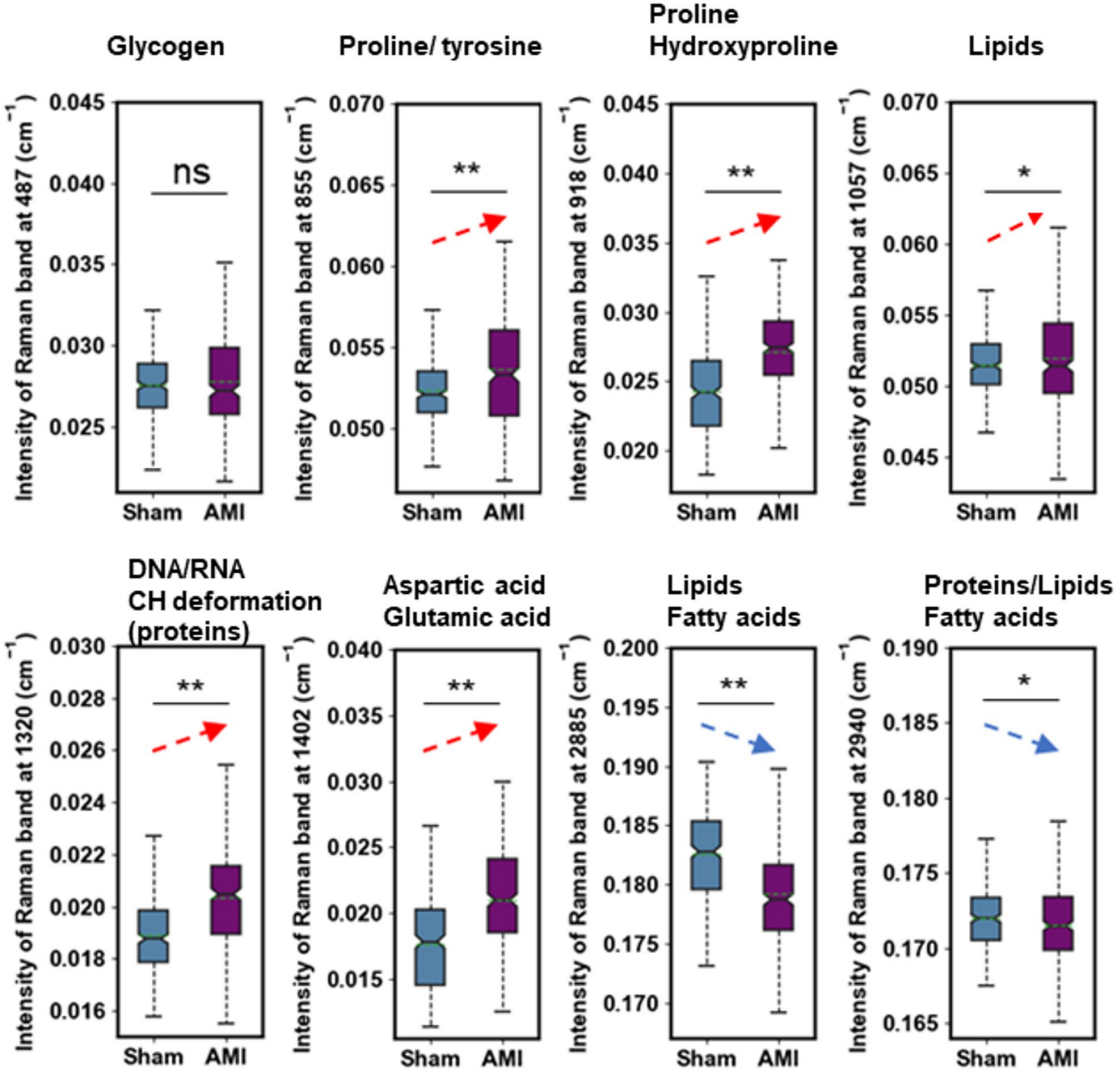
Quantitative analysis of differential Raman peaks between two groups. Differential Raman spectrum between AMI (purple) from Sham (blue) group are presented in box plots. Elevated Raman spectrum in AMI group including proline or tyrosine (855 cm^− 1^), proline or hydroxyproline (918 cm^− 1^), G (DNA/RNA) or CH deformation (1320 cm^− 1^) and amino acids aspartic & glutamic acid (1402 cm^− 1^). and those downregulated in AMI group including (2885 cm^− 1^ and 2940 cm^− 1^). * represents p value < 0.05, and ** represents *p* value < 0.01.

Quantitative analysis revealed elevated intensities of several Raman peaks associated with amino acids in the AMI group. These included peaks assigned to proline or tyrosine (855 cm^− 1^), proline or hydroxyproline (918 cm^− 1^), G (DNA/RNA) or CH deformation (1320 cm^− 1^) and amino acids aspartic & glutamic acid (1402 cm^− 1^). In contrast, the majority of lipid and fatty acid peaks, specifically at 2885 cm^− 1^ and 2940 cm^− 1^, which showed higher intensities in the Sham group (Fig. 4).

### Machine learning-based diagnostic model for AMI

To classify plasma samples from the AMI group using Raman spectra, we employed five machine learning algorithms: SVM, LR, RF, LDA, and PLS-DA. A plasma Raman spectral database was constructed from 27 AMI and 27 Sham mice, with 12 spectra for each plasma sample, yielding in a total of 648 spectra. All spectra covered a range of 400 to 3000 cm^− 1^. Prior to analysis, the spectral data were preprocessed by baseline subtraction and smoothing.

**Fig. 5:**
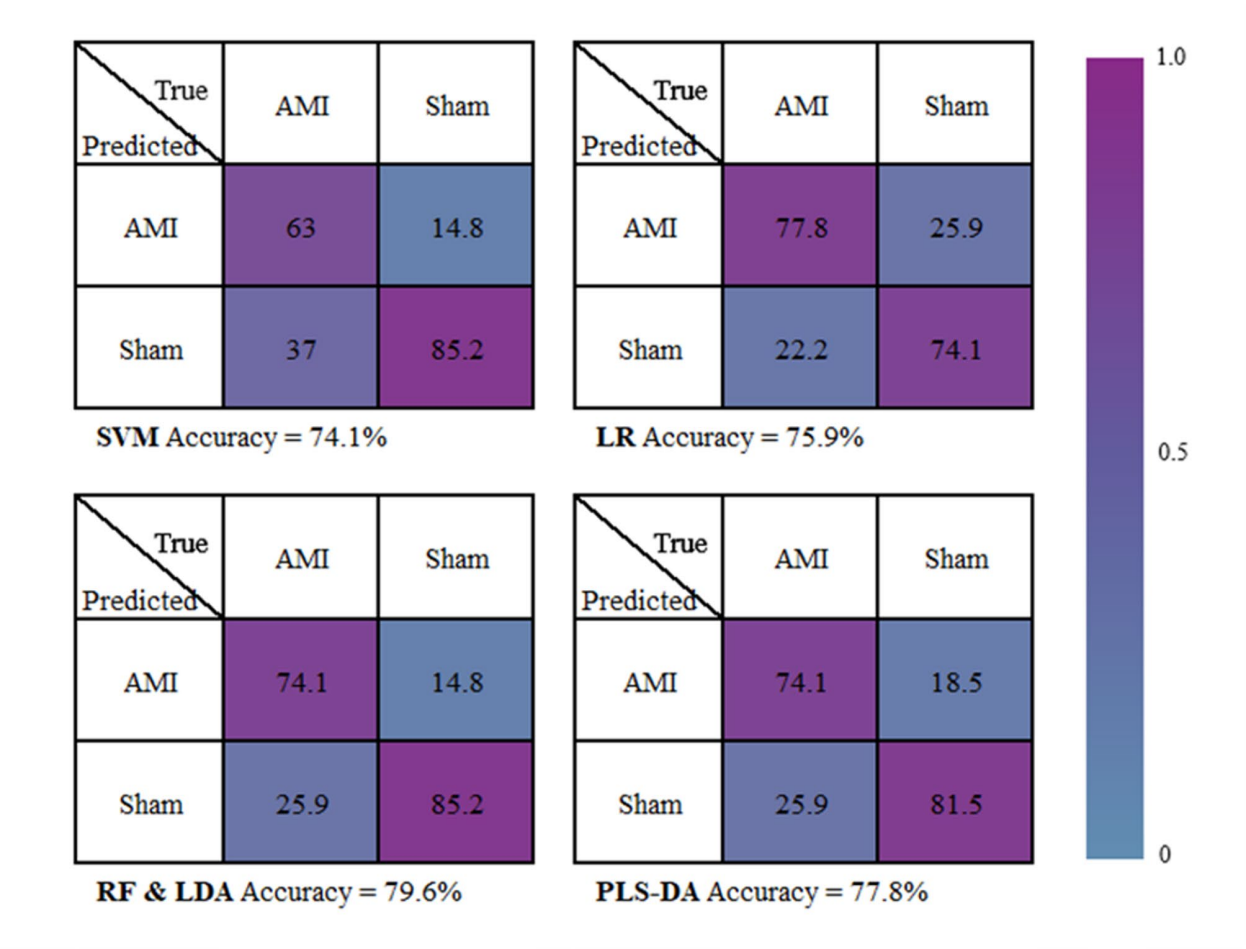
AMI model construction based on machine learning algorithms. AMI model construction by binary confusion matrices for classifying Raman spectra of each mouse in AMI and Sham groups, five machine learning algorithms, expressed as percentage (%).

As shown in Fig. 5 showed that both RF and LDA achieved the highest classification accuracy (79.6%) among all evaluated algorithms. indicating their strong reliability for this classification tasks. These two algorithms also exhibited a high specificity (85.2%) and a moderate sensitivity (74.1%), demonstrating their effectiveness in distinguishing between AMI and Sham groups. PLS-DA performed satisfactorily with accuracy of 77.8%, a specificity of 81.5%, and a sensitivity of 74.1%, which suggests its competence in managing classification tasks. LR displayed a commendable balance across the metrics (accuracy:75.9%; specificity:74.1%; sensitivity:77.8%). In contrast, SVM showed the lowest performance with an accuracy of 74.1%, specificity of 85.2%, and sensitivity of 63.0%. The findings indicated that both RF and LDA demonstrated superior accuracy in differentiating AMI from the Sham group among the five algorithms evaluated.

### Metabolomics analysis of plasma samples

**Fig. 6:**
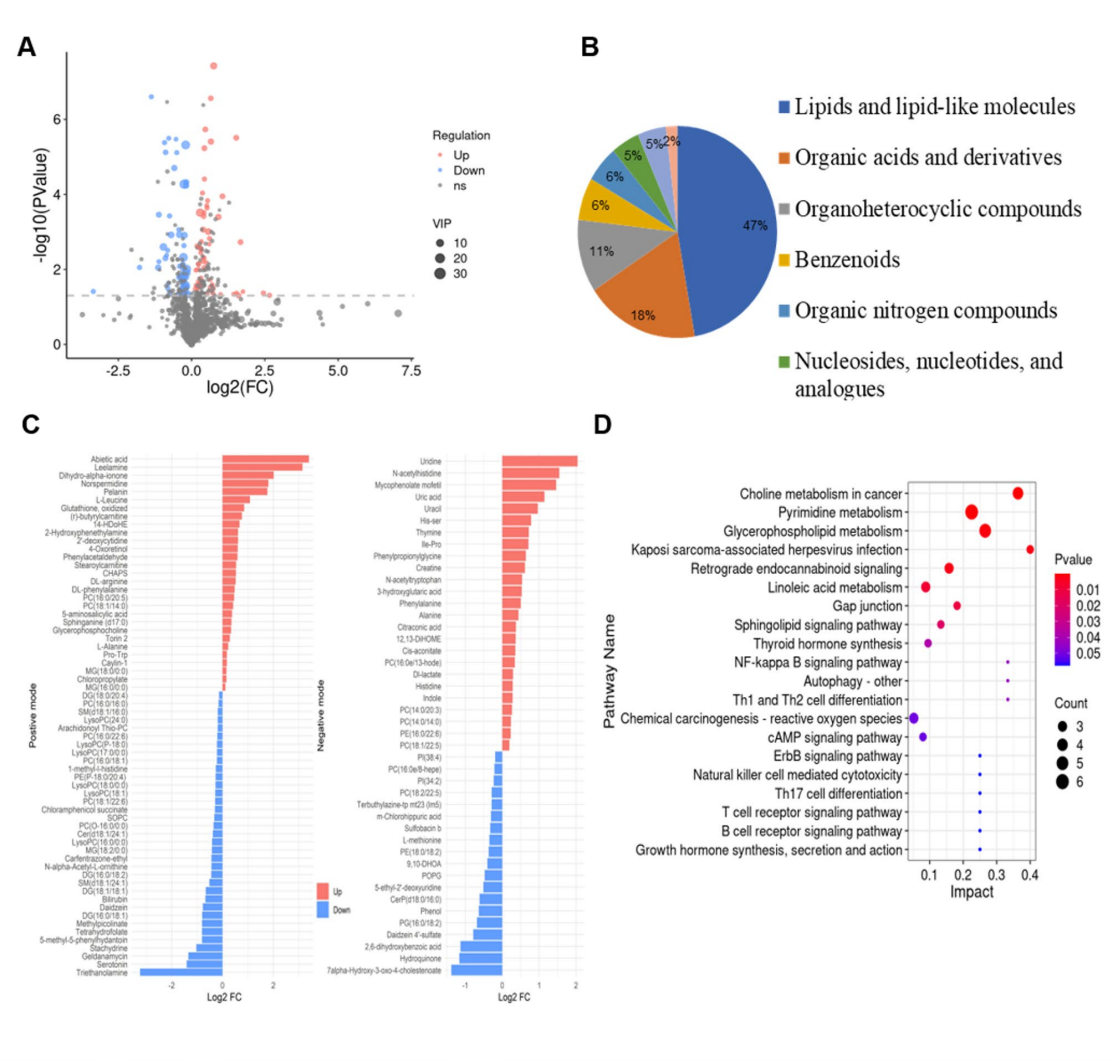
Non-targeted metabolomic analysis of mice plasma. All mice plasma were subjected to non-targeted metabolites analysis via MS. **(A)** The volcano plot of 108 differential metabolites identified. **(B)** A pie chart categorizing the classification of differential metabolites. **(C)** The histogram of differential metabolites in ESI^+^ and ESI^−^ modes. The color red indicates up regulation, while the color blue signifies down regulation. **(D)** KEGG enrichment analysis of differential metabolites.

To further validate alterations in the biochemical composition of plasma samples, we performed a non-targeted metabolomics approach utilizing Triple Time-of-Flight Mass Spectrometry (UPLC-Triple-TOF-MS). A total of 54 plasma samples-27 from AMI group and 27 from Sham group-were included in this study. A total of 840 and 627 raw features were respectively filtered and extracted in positive and negative ion (ESI^+^ and ESI^−^) modes, respectively. OPLS-DA was utilized to illustrate metabolic disturbances associated with AMI. The OPLS-DA model demonstrated both stable and reliable in Supplementary Fig. 2. Using a threshold of VIP > 1 and *p* < 0.05, we identified 108 differential metabolites (Fig. 6A), comprising 64 metabolites in ESI^+^ mode and 44 in ESI^−^ mode (Supplementary Table 1).

**Fig. 7:**
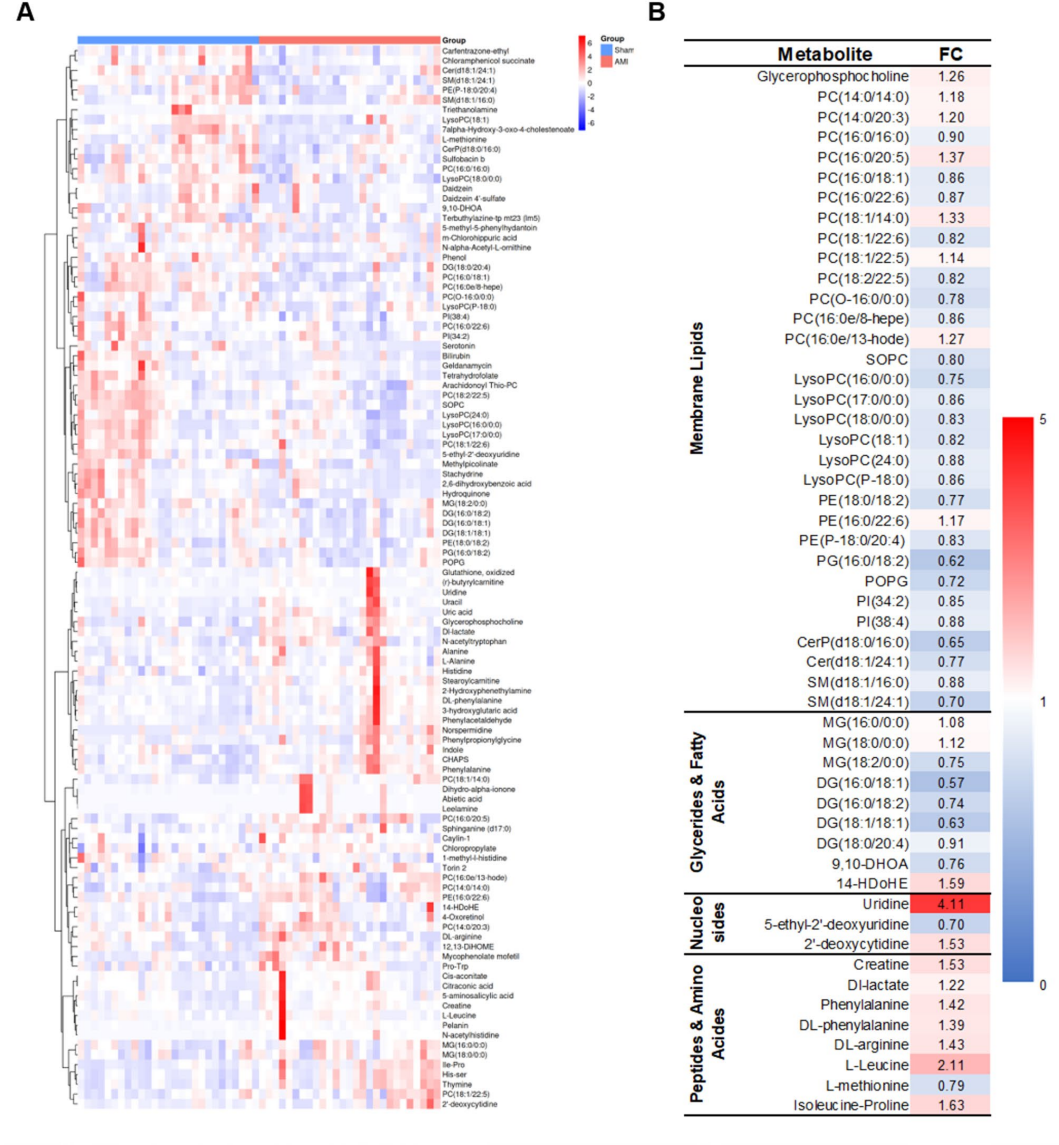
Analysis of differential metabolites. Metabolomic difference in mice plasma metabolites were identified by non-targeted MS. **(A)** The heatmap of differential expressed metabolites in. **(B)** The fold change (FC) differential metabolites between two groups. The differential expressed metabolites including the classes of membrane lipids, glycerides & fatty acids, nucleic acids, and peptides & amino acids. The following abbreviations are utilized: PC for phosphatidylcholine, PE for phosphatidylethanolamine, PG for phosphatidylglycerol, SM for sphingomyelin, MG for monoradylglycerol, and DG for diacylglycerol.

These metabolites were categorized into eight major classes (Fig. 6B), with lipids and lipid-like molecules, organic acids and derivatives, and organoheterocyclic compounds being the three predominant categories. Notably, lipids and lipid-like molecules accounted for 47% of the identified metabolites, consistent with the lipid profiles observed in Raman intensity analyses. In ESI^+^ mode, 29 metabolites were upregulated while 35 were downregulated (Figs. 6C and 7A). In ESI^−^ mode, 25 metabolites were upregulated and 19 were downregulated in AMI group compared to Sham groups. KEGG enrichment analysis revealed the top five pathways among 108 differentially enriched metabolites (Fig. 6D): Choline metabolism in cancer, Pyrimidine metabolism, Glycerophospholipid metabolism, Retrograde endocannabinoid signaling, and Kaposi sarcoma-associated herpesvirus infection.

As illustrated in Fig. 7B, a substantial proportion of membrane phospholipid species, such as PC (16:0/16:0), PC (16:0/18:1), PC (18:2/22:5), SOPC, LysoPC, PE (18:0/18:2), PE (P-18:0/20:4), PG (16:0), POPG, PI, CerP (d18:0/16:0), Cer (d18:1/24:1), SM (d18:1/16:0), and SM (d18:1/24:1), showed downregulated expression in AMI group, corroborating the reduction in lipid-assigned Raman signal (2885 cm ^− 1^ and 2940 cm^− 1^) observed in Raman spectra, whereas 1407 cm^− 1^ exhibited an opposite trend. Similarly, a majority of membrane glycerides and fatty acid species, including MG (18:2/0:0), DG (16:0/18:1), DG (16:0/18:2), DG (18:1/18:1), DG (18:0/20:4), and 9,10-DHOA, were downregulated, further supporting the Raman-based observation of reduced lipid/fatty acid (2885 cm^− 1^ and 2940 cm^− 1^) content. Within the nucleic acid category, uridine exhibited a significant upregulation in the AMI group that consistent with 1320 cm^− 1^ (DNA/RNA/CH deformation). Conversely, proline and tyrosine, which showed elevated signal in Raman spectra, did not show significant changes in expression levels in the metabolomic data. Several peptides and amino acids, including creatine, DL-lactate, phenylalanine, DL-phenylalanine, DL-arginine, L-leucine, L-methionine, and isoleucine-proline, were upregulated.

## Discussion

This study developed and validated a novel diagnostic model for AMI by integrating Raman spectroscopy with machine learning algorithms. The combination of Raman spectroscopy, which provides unique metabolic fingerprinting capabilities, and machine learning, which offers robust classification power, enabled the development of a rapid and accurate diagnostic approach for AMI.

To ensure homogeneity and minimize confounding factors, we developed murine models of AMI and subsequently collected plasma spectra. Our findings revealed eight distinct Raman peaks that effectively differentiated plasma samples from the AMI and Sham groups Spectral assignments indicated that lipids and fatty acids play crucial roles in AMI pathophysiology. Previous research has suggested that particular types of ceramides and their respective ratios may serve as potential risk indicators for patients experiencing AMI^34^. Additionally, the plasma sphingomyelin-to-acid ceramidase ratio has been proposed as a novel biomarker for therapeutic strategies aimed at preventing adverse remodeling following myocardial infarction^35^. Alterations in glycerophospholipid metabolism and arginine biosynthesis have been implicated in the pathogenesis and progression of AMI^36^. Various lipids, such as phosphatidylcholine, lysophosphatidylcholine and phosphatidylethanolamine, may serve as predictive biomarkers for predicting mortality and heart failure following AMI^37^. We observed an apparent contradiction in our integrated metabolomics analysis: while the majority of lipid categories showed significantly downregulated expression in MS data, among the three Raman peaks assigned to lipids (2885 cm ^− 1^,2940 cm^− 1^ and 1407 cm^− 1^), 2885 cm ^− 1^,2940 cm^− 1^ were decreased significantly and 1407 cm^− 1^ was strikingly increased (Figs. 3 and 6). Several plausible explanations may account for this discrepancy: First, Raman spectroscopy captures the overall spectral profile of lipids, whereas MS identifies and quantifies specific molecular species. Metabolites that did not reach statistical significance in MS were excluded from the differential analysis, which may lead to an incomplete representation of lipid dynamics. Second, the decrease in two of the three lipid-associated Raman peaks still reflects a general declining trend, which is consistent with the overall reduction in membrane lipids detected via MS. Third, lipids comprise a highly diverse group of compounds, including simple lipids, complex lipids (such as phospholipids and glycolipids), and derived lipids (including fatty acids and sterols). The MS results revealed alterations across multiple lipid subclasses, such as phosphatidylcholines, sphingomyelins, and triglycerides. Thus, the Raman spectroscopy-based lipid profile is, to some extent, consistent with the MS-derived lipidomic profile. This discrepancy between Raman spectroscopy and MS were also observed aspartic acid glutamic acid (Figs. 3 and 6). These findings demonstrate that the combined use of Raman spectroscopy and mass spectrometry offers a promising methodological framework for future research. Their complementary nature supports potential applications in rapid clinical diagnostics, especially where high-throughput and label-free screening is desirable. Furthermore, we employed five machine learning algorithms including SVM, LR, RF, LDA and PLS-DA, to classify plasma samples from the AMI and Sham groups. Both RF and LDA demonstrated the highest performance, achieving an accuracy of 79.6%, specificity of 85.2%, and sensitivity of 74.1%. These results underscore the potential of integrating Raman spectroscopy with advanced machine learning algorithms such as RF and LDA for the early AMI diagnosis.

Several limitations of this study should be acknowledged. Firstly, the relatively small sample size may affect the predictive accuracy and generalizability of the machine learning models employed. Secondly, the precise correlation between Raman spectra and specific metabolites requires further elucidation. Third, potential confounding effects of anesthetics or future clinical medications on Raman spectra should be considered in subsequent studies. More advanced artificial intelligence methods may help mitigate this effect and improve the model generalizability.

In conclusion, this study established a rapid diagnostic model for AMI using Raman spectroscopy-based metabolic profiling in a murine model. With an accuracy of 79.6%, this model indicates potential applications of AMI diagnosis or other emergency medical conditions.

## Supplementary Information

Below is the link to the electronic supplementary material.

Supplementary Material 1

Supplementary Material 2

Supplementary Material 3

## Data Availability

The data supporting the findings of this study are available from the corresponding author upon reasonable request.

## References

[1] Reed, G. W., Rossi, J. E. & Cannon, C. P. Acute myocardial infarction. *Lancet* **389**, 197–210. 10.1016/S0140-6736(16)30677-8 (2017). 27502078 10.1016/S0140-6736(16)30677-8

[2] Thygesen, K. et al. Fourth universal definition of myocardial infarction (2018). *J. Am. Coll. Cardiol.* **72**, 2231–2264. 10.1016/j.jacc.2018.08.1038 (2018). 30153967 10.1016/j.jacc.2018.08.1038

[3] Hsieh, Y. K. et al. Recent advances in the diagnosis and management of acute myocardial infarction. *J. Chin. Med. Assoc.* **86**, 950–959. 10.1097/JCMA.0000000000001001 (2023). 37801590 10.1097/JCMA.0000000000001001 PMC12718803

[4] Apple, F. S., Sandoval, Y., Jaffe, A. S., Ordonez-Llanos, J. & Bio-Markers I. T. F. o. C. A. o. C. Cardiac troponin assays: guide to Understanding analytical characteristics and their impact on clinical care. *Clin. Chem.* **63**, 73–81. 10.1373/clinchem.2016.255109 (2017). 28062612 10.1373/clinchem.2016.255109

[5] Liebetrau, C. et al. Release kinetics of cardiac biomarkers in patients undergoing Transcoronary ablation of septal hypertrophy. *Clin. Chem.* **58**, 1049–1054. 10.1373/clinchem.2011.178129 (2012). 22504118 10.1373/clinchem.2011.178129

[6] Ibanez, B. et al. 2017 ESC guidelines for the management of acute myocardial infarction in patients presenting with ST-segment elevation: the task force for the management of acute myocardial infarction in patients presenting with ST-segment elevation of the European society of cardiology (ESC). *Eur. Heart J.* **39**, 119–177. 10.1093/eurheartj/ehx393 (2018). 28886621 10.1093/eurheartj/ehx393

[7] Zeitouni, M. et al. Clinical outcomes according to ECG presentations in infarct-related cardiogenic shock in the culprit lesion only PCI vs multivessel PCI in cardiogenic shock trial. *Chest* **159**, 1415–1425. 10.1016/j.chest.2020.10.089 (2021). 33248059 10.1016/j.chest.2020.10.089

[8] Gallacher, P. J. et al. High-sensitivity cardiac troponin and the diagnosis of myocardial infarction in patients with kidney impairment. *Kidney Int.* **102**, 149–159. 10.1016/j.kint.2022.02.019 (2022). 35271932 10.1016/j.kint.2022.02.019

[9] Ammirati, E. et al. Management of acute myocarditis and chronic inflammatory cardiomyopathy: An expert consensus document. *Circ. Heart Fail.* **13**, e007405. 10.1161/CIRCHEARTFAILURE.120.007405 (2020). 33176455 10.1161/CIRCHEARTFAILURE.120.007405 PMC7673642

[10] Boeddinghaus, J. et al. High-sensitivity cardiac troponin I assay for early diagnosis of acute myocardial infarction. *Clin. Chem.* **65**, 893–904. 10.1373/clinchem.2018.300061 (2019). 30988172 10.1373/clinchem.2018.300061

[11] Byrne, R. A. et al. 2023 ESC guidelines for the management of acute coronary syndromes. *Eur. Heart J.* **44**, 3720–3826. 10.1093/eurheartj/ehad191 (2023). 37622654 10.1093/eurheartj/ehad191

[12] Ajoolabady, A. et al. Implication in cardiovascular research and diseases. *Obes. Rev.* **25**, e13825. 10.1111/obr.13825 (2024). 39370721 10.1111/obr.13825

[13] Griffin, J. L., Atherton, H., Shockcor, J. & Atzori, L. Metabolomics as a tool for cardiac research. *Nat. Rev. Cardiol.* **8**, 630–643. 10.1038/nrcardio.2011.138 (2011). 21931361 10.1038/nrcardio.2011.138

[14] Khan, A. et al. High-resolution metabolomics study revealing l-homocysteine sulfinic acid, cysteic acid, and carnitine as novel biomarkers for high acute myocardial infarction risk. *Metabolism* **104**, 154051. 10.1016/j.metabol.2019.154051 (2020). 31874143 10.1016/j.metabol.2019.154051

[15] Guo, M. et al. Targeted metabolomic analysis of plasma fatty acids in acute myocardial infarction in young adults. *Nutr. Metab. Cardiovasc. Dis.* **31**, 3131–3141. 10.1016/j.numecd.2021.06.024 (2021). 34625358 10.1016/j.numecd.2021.06.024

[16] Zhou, J. et al. Metabolomics analysis identifies differential metabolites as biomarkers for acute myocardial infarction. *Biomolecules* **14**10.3390/biom14050532 (2024). 10.3390/biom14050532 PMC11117998 38785939

[17] Kong, K., Kendall, C., Stone, N. & Notingher, I. Raman spectroscopy for medical diagnostics–From in-vitro biofluid assays to in-vivo cancer detection. *Adv. Drug Deliv Rev.* **89**, 121–134. 10.1016/j.addr.2015.03.009 (2015). 25809988 10.1016/j.addr.2015.03.009

[18] Ho, C. S. et al. Rapid identification of pathogenic bacteria using Raman spectroscopy and deep learning. *Nat. Commun.* **10**, 4927. 10.1038/s41467-019-12898-9 (2019). 31666527 10.1038/s41467-019-12898-9 PMC6960993

[19] Ralbovsky, N. M. & Lednev, I. K. Towards development of a novel universal medical diagnostic method: Raman spectroscopy and machine learning. *Chem. Soc. Rev.* **49**, 7428–7453. 10.1039/d0cs01019g (2020). 32996518 10.1039/d0cs01019g

[20] Huang, L. et al. Noninvasive diagnosis of gastric cancer based on breath analysis with a tubular Surface-Enhanced Raman scattering sensor. *ACS Sens.* **7**, 1439–1450. 10.1021/acssensors.2c00146 (2022). 35561250 10.1021/acssensors.2c00146

[21] Wang, Y., Fang, L., Wang, Y. & Xiong, Z. Current trends of Raman spectroscopy in clinic settings: Opportunities and challenges. *Adv. Sci. (Weinh)*. **11**, e2300668. 10.1002/advs.202300668 (2024). 38072672 10.1002/advs.202300668 PMC10870035

[22] Cutshaw, G. et al. Metabolic response to small molecule therapy in colorectal cancer tracked with Raman spectroscopy and metabolomics. *Angew Chem. Int. Ed. Engl.* **63**, e202410919. 10.1002/anie.202410919 (2024). 38995663 10.1002/anie.202410919 PMC11473224

[23] Gao, E. et al. A novel and efficient model of coronary artery ligation and myocardial infarction in the mouse. *Circ. Res.* **107**, 1445–1453. 10.1161/CIRCRESAHA.110.223925 (2010). 20966393 10.1161/CIRCRESAHA.110.223925 PMC3005817

[24] Rohleder, D., Kiefer, W. & Petrich, W. Quantitative analysis of serum and serum ultrafiltrate by means of Raman spectroscopy. *Analyst* **129**, 906–911. 10.1039/b408927h (2004). 15457321 10.1039/b408927h

[25] Kanehisa, M., Furumichi, M., Sato, Y., Matsuura, Y. & Ishiguro-Watanabe, M. KEGG: Biological systems database as a model of the real world. *Nucleic Acids Res.* **53**, D672–D677. 10.1093/nar/gkae909 (2025). 39417505 10.1093/nar/gkae909 PMC11701520

[26] Stone, N., Kendall, C., Smith, J., Crow, P. & Barr, H. Raman spectroscopy for identification of epithelial cancers. *Faraday Discuss.* **126**, 141–157. 10.1039/b304992b (2004). discussion 169–183. 14992404 10.1039/b304992b

[27] Huang, Z. et al. Near-infrared Raman spectroscopy for optical diagnosis of lung cancer. *Int. J. Cancer*. **107**, 1047–1052. 10.1002/ijc.11500 (2003). 14601068 10.1002/ijc.11500

[28] Cheng, W. T., Liu, M. T., Liu, H. N. & Lin, S. Y. Micro-Raman spectroscopy used to identify and grade human skin Pilomatrixoma. *Microsc. Res. Tech.* **68**, 75–79. 10.1002/jemt.20229 (2005). 16228983 10.1002/jemt.20229

[29] Saleem, H. et al. FT-Raman spectral and conformational studies on (E)-2-(2-hydroxybenzylidenamino)-3-(1H-indol-3yl) propionic acid. *Spectrochim Acta Mol. Biomol. Spectrosc.* **101**, 91–99. 10.1016/j.saa.2012.09.023 (2013). 10.1016/j.saa.2012.09.023 23099165

[30] Ralbovsky, N. M. & Lednev, I. K. Vibrational spectroscopy for detection of diabetes: A review. *Appl. Spectrosc.* **75**, 929–946. 10.1177/00037028211019130 (2021). 33988040 10.1177/00037028211019130

[31] Notingher, I. et al. Discrimination between ricin and sulphur mustard toxicity in vitro using Raman spectroscopy. *J. R Soc. Interface*. **1**, 79–90. 10.1098/rsif.2004.0008 (2004). 16849154 10.1098/rsif.2004.0008 PMC1618929

[32] Shetty, G., Kendall, C., Shepherd, N., Stone, N. & Barr, H. Raman spectroscopy: Elucidation of biochemical changes in carcinogenesis of oesophagus. *Br. J. Cancer*. **94**, 1460–1464. 10.1038/sj.bjc.6603102 (2006). 16622450 10.1038/sj.bjc.6603102 PMC2361283

[33] Wang, S. et al. Controlling cross pumping between C-N and C-H vibration in nitromethane by selective fluorescence-enhanced stimulated Raman scattering. *Opt. Express*. **24**, 10132–10141. 10.1364/OE.24.010132 (2016). 27137622 10.1364/OE.24.010132

[34] Michelucci, E. et al. Ceramides and cardiovascular risk Factors, inflammatory parameters and left ventricular function in AMI patients. *Biomedicines* **10**10.3390/biomedicines10020429 (2022). 10.3390/biomedicines10020429 PMC8962314 35203637

[35] Huang, C. H. et al. Sphingolipid metabolism is associated with cardiac dyssynchrony in patients with acute myocardial infarction. *Biomedicines* **12** (2024). 10.3390/biomedicines12081864 10.3390/biomedicines12081864 PMC11351212 39200328

[36] Fu, M. et al. Multinomial machine learning identifies independent biomarkers by integrated metabolic analysis of acute coronary syndrome. *Sci. Rep.* **13**, 20535. 10.1038/s41598-023-47783-5 (2023). 37996510 10.1038/s41598-023-47783-5 PMC10667512

[37] Guo, C. et al. Lipidomic analyses reveal potential biomarkers for predicting death and heart failure after acute myocardial infarction. *Clin. Chim. Acta*. **562**, 119892. 10.1016/j.cca.2024.119892 (2024). 39068962 10.1016/j.cca.2024.119892

