# Supplementary material for: Plasma-based Raman spectroscopy for early detection of acute myocardial infarction in murine models
Source: Sci Rep. 2025 Dec 10;16:705. doi: 10.1038/s41598-025-30292-y (PMC12780145; doi:10.1038/s41598-025-30292-y)
Supplement: Supplementary file 1 — Supplementary Material 1 [file 41598_2025_30292_MOESM1_ESM.docx]

**Supplementary Table 1. Metabolites with significant differences between the AMI and sham groups.**

| **id** | **Metabolite** | **Mode** | **RT(s)** | **Extract mass** | **FC** | **VIP** | ***P* value** |
| --- | --- | --- | --- | --- | --- | --- | --- |
| 1 | Hydroquinone | ESI- | 24.49 | 109.0289 | 0.45 | 3.53 | 0.001 |
| 2 | 2,6-dihydroxybenzoic acid | ESI- | 26.24 | 153.0185 | 0.46 | 3.62 | 0.004 |
| 3 | Phenol | ESI- | 25.44 | 93.0343 | 0.64 | 5.79 | 0.004 |
| 4 | Citraconic acid | ESI- | 423.35 | 129.0186 | 1.29 | 1.27 | 0.040 |
| 5 | 12,13-DiHOME | ESI- | 78.98 | 313.2365 | 1.29 | 1.57 | 0.015 |
| 6 | 9,10-DHOA | ESI- | 98.87 | 313.2365 | 0.76 | 1.54 | 0.106 |
| 7 | Sulfobacin b | ESI- | 30.89 | 574.4484 | 0.80 | 3.97 | 0.012 |
| 8 | PC(14:0/14:0) | ESI- | 135.51 | 722.5097 | 1.18 | 1.90 | 0.012 |
| 9 | PC(14:0/20:3) | ESI- | 138.58 | 814.5570 | 1.20 | 1.63 | 0.015 |
| 10 | PC(18:1/22:5) | ESI- | 131.77 | 892.6027 | 1.14 | 1.72 | 0.024 |
| 11 | PC(18:2/22:5) | ESI- | 132.28 | 890.5876 | 0.82 | 1.49 | 0.003 |
| 12 | PC(16:0e/8-hepe) | ESI- | 134.91 | 840.5735 | 0.86 | 3.90 | <0.001 |
| 13 | PC(16:0e/13-hode) | ESI- | 77.74 | 818.5860 | 1.27 | 3.04 | 0.016 |
| 14 | PE(18:0/18:2) | ESI- | 103.04 | 742.5360 | 0.77 | 1.23 | 0.039 |
| 15 | PE(16:0/22:6) | ESI- | 134.78 | 746.5106 | 1.17 | 2.72 | 0.029 |
| 16 | PG(16:0/18:2) | ESI- | 78.13 | 745.4998 | 0.62 | 1.57 | <0.001 |
| 17 | POPG | ESI- | 78.57 | 747.5145 | 0.72 | 1.24 | 0.003 |
| 18 | PI(34:2) | ESI- | 186.39 | 833.5161 | 0.85 | 4.24 | 0.132 |
| 19 | PI(38:4) | ESI- | 182.28 | 885.5490 | 0.88 | 13.54 | 0.009 |
| 20 | CerP(d18:0/16:0) | ESI- | 33.72 | 618.4742 | 0.65 | 4.28 | <0.001 |
| 21 | 7alpha-Hydroxy-3-oxo-4-cholestenoate | ESI- | 75.79 | 429.2981 | 0.38 | 1.66 | <0.001 |
| 22 | Ile-Pro | ESI- | 112.77 | 227.0661 | 1.63 | 4.68 | <0.001 |
| 23 | His-ser | ESI- | 100.24 | 241.0818 | 1.72 | 7.08 | <0.001 |
| 24 | Uridine | ESI- | 156.88 | 243.0609 | 4.11 | 3.23 | 0.065 |
| 25 | 5-ethyl-2'-deoxyuridine | ESI- | 103.23 | 255.1013 | 0.70 | 1.27 | 0.041 |
| 26 | Creatine | ESI- | 341.10 | 130.0615 | 1.53 | 1.09 | 0.012 |
| 27 | 3-hydroxyglutaric acid | ESI- | 259.36 | 147.0443 | 1.44 | 1.21 | 0.004 |
| 28 | L-methionine | ESI- | 298.04 | 148.0430 | 0.79 | 1.80 | 0.057 |
| 29 | Histidine | ESI- | 393.10 | 154.0614 | 1.22 | 1.43 | 0.036 |
| 30 | Phenylalanine | ESI- | 260.06 | 164.0708 | 1.42 | 3.21 | <0.001 |
| 31 | Cis-aconitate | ESI- | 423.27 | 173.0082 | 1.28 | 1.24 | 0.051 |
| 32 | N-acetylhistidine | ESI- | 307.33 | 196.0717 | 2.91 | 1.51 | 0.104 |
| 33 | Phenylpropionylglycine | ESI- | 177.50 | 206.0811 | 1.55 | 1.01 | 0.010 |
| 34 | N-acetyltryptophan | ESI- | 183.95 | 245.0918 | 1.45 | 1.62 | 0.002 |
| 35 | Alanine | ESI- | 340.83 | 88.0400 | 1.36 | 1.09 | 0.016 |
| 36 | Dl-lactate | ESI- | 218.15 | 89.0249 | 1.22 | 5.12 | 0.041 |
| 37 | Uracil | ESI- | 88.13 | 111.0194 | 1.95 | 4.68 | 0.006 |
| 38 | Indole | ESI- | 297.26 | 116.0710 | 1.21 | 1.67 | 0.029 |
| 39 | Thymine | ESI- | 100.58 | 125.0350 | 1.65 | 2.84 | <0.001 |
| 40 | Uric acid | ESI- | 323.16 | 167.0201 | 2.21 | 3.14 | 0.002 |
| 41 | Terbuthylazine-tp mt23 (lm5) | ESI- | 103.10 | 183.1019 | 0.82 | 1.33 | 0.063 |
| 42 | Mycophenolate mofetil | ESI- | 34.09 | 432.2188 | 2.75 | 3.55 | 0.001 |
| 43 | Daidzein 4'-sulfate | ESI- | 26.26 | 333.0053 | 0.58 | 2.15 | 0.069 |
| 44 | m-Chlorohippuric acid | ESI- | 151.75 | 213.0159 | 0.81 | 1.46 | 0.053 |
| 45 | Phenylacetaldehyde | ESI+ | 249.12 | 103.0531 | 1.50 | 1.92 | 0.003 |
| 46 | 5-aminosalicylic acid | ESI+ | 335.15 | 136.0472 | 1.30 | 1.43 | 0.027 |
| 47 | Chloropropylate | ESI+ | 370.45 | 361.0191 | 1.11 | 1.93 | 0.025 |
| 48 | Chloramphenicol succinate | ESI+ | 296.56 | 445.0296 | 0.81 | 2.43 | 0.008 |
| 49 | (r)-butyrylcarnitine | ESI+ | 245.24 | 232.1550 | 1.70 | 11.04 | 0.061 |
| 50 | MG(18:2/0:0) | ESI+ | 31.63 | 263.2354 | 0.75 | 1.72 | 0.001 |
| 51 | 14-HDoHE | ESI+ | 51.71 | 327.2301 | 1.59 | 1.12 | 0.064 |
| 52 | Stearoylcarnitine | ESI+ | 159.71 | 428.3703 | 1.44 | 2.01 | 0.004 |
| 53 | DG(16:0/18:2) | ESI+ | 30.24 | 615.4939 | 0.74 | 1.17 | 0.001 |
| 54 | DG(18:1/18:1) | ESI+ | 30.06 | 634.5371 | 0.63 | 2.03 | 0.008 |
| 55 | MG(16:0/0:0) | ESI+ | 31.47 | 313.2731 | 1.08 | 1.31 | 0.103 |
| 56 | MG(18:0/0:0) | ESI+ | 32.03 | 341.3037 | 1.12 | 1.29 | 0.024 |
| 57 | DG(16:0/18:1) | ESI+ | 30.13 | 617.5105 | 0.57 | 2.04 | 0.001 |
| 58 | DG(18:0/20:4) | ESI+ | 181.49 | 627.5325 | 0.91 | 4.04 | 0.123 |
| 59 | Glycerophosphocholine | ESI+ | 260.08 | 104.1069 | 1.26 | 11.99 | <0.001 |
| 60 | PC(16:0/16:0) | ESI+ | 139.37 | 734.5664 | 0.90 | 3.90 | 0.002 |
| 61 | PC(16:0/20:5) | ESI+ | 129.94 | 766.5712 | 1.37 | 1.80 | 0.001 |
| 62 | PC(16:0/18:1) | ESI+ | 133.05 | 782.5695 | 0.86 | 14.15 | <0.001 |
| 63 | PC(16:0/22:6) | ESI+ | 131.54 | 806.5700 | 0.87 | 15.05 | 0.006 |
| 64 | PC(18:1/14:0) | ESI+ | 138.74 | 732.5499 | 1.33 | 3.49 | 0.001 |
| 65 | PC(18:1/22:6) | ESI+ | 128.55 | 818.6021 | 0.82 | 2.08 | 0.003 |
| 66 | PC(O-16:0/0:0) | ESI+ | 187.16 | 482.3568 | 0.78 | 4.39 | 0.015 |
| 67 | SOPC | ESI+ | 129.89 | 832.5841 | 0.80 | 5.81 | <0.001 |
| 68 | LysoPC(16:0/0:0) | ESI+ | 177.13 | 538.3834 | 0.75 | 4.41 | 0.026 |
| 69 | LysoPC(17:0/0:0) | ESI+ | 180.29 | 510.3527 | 0.86 | 4.33 | 0.014 |
| 70 | LysoPC(18:0/0:0) | ESI+ | 176.51 | 568.3392 | 0.83 | 16.75 | <0.001 |
| 71 | LysoPC(18:1) | ESI+ | 177.76 | 544.3393 | 0.82 | 13.88 | 0.021 |
| 72 | LysoPC(24:0) | ESI+ | 172.06 | 608.4618 | 0.88 | 2.09 | 0.020 |
| 73 | LysoPC(P-18:0) | ESI+ | 183.98 | 508.3729 | 0.86 | 1.99 | 0.097 |
| 74 | PE(P-18:0/20:4) | ESI+ | 130.02 | 752.5548 | 0.83 | 1.01 | 0.009 |
| 75 | Leelamine | ESI+ | 31.54 | 173.1311 | 8.86 | 2.11 | 0.061 |
| 76 | Abietic acid | ESI+ | 30.68 | 257.2249 | 10.53 | 2.26 | 0.069 |
| 77 | 4-Oxoretinol | ESI+ | 52.21 | 301.2142 | 1.51 | 1.10 | 0.032 |
| 78 | Cer(d18:1/24:1) | ESI+ | 31.81 | 630.6151 | 0.77 | 1.92 | 0.046 |
| 79 | SM(d18:1/16:0) | ESI+ | 168.99 | 703.5757 | 0.88 | 15.27 | 0.092 |
| 80 | SM(d18:1/24:1) | ESI+ | 187.77 | 813.6821 | 0.70 | 6.77 | <0.001 |
| 81 | Pelanin | ESI+ | 531.24 | 357.2474 | 3.39 | 1.27 | 0.085 |
| 82 | CHAPS | ESI+ | 158.51 | 448.3394 | 1.44 | 1.59 | <0.001 |
| 83 | 2'-deoxycytidine | ESI+ | 196.89 | 455.1873 | 1.53 | 1.90 | 0.009 |
| 84 | DL-arginine | ESI+ | 70.49 | 130.0851 | 1.43 | 2.12 | 0.128 |
| 85 | L-Leucine | ESI+ | 325.16 | 132.1006 | 2.11 | 1.14 | 0.104 |
| 86 | L-Alanine | ESI+ | 335.83 | 134.0178 | 1.17 | 1.35 | 0.093 |
| 87 | Stachydrine | ESI+ | 264.38 | 144.1012 | 0.49 | 8.99 | 0.006 |
| 88 | N-alpha-Acetyl-L-ornithine | ESI+ | 284.51 | 157.0961 | 0.74 | 1.04 | 0.185 |
| 89 | DL-phenylalanine | ESI+ | 249.43 | 166.0860 | 1.39 | 3.71 | 0.031 |
| 90 | 1-methyl-l-histidine | ESI+ | 413.29 | 170.0918 | 0.83 | 2.50 | 0.034 |
| 91 | Tetrahydrofolate | ESI+ | 171.60 | 299.1376 | 0.57 | 1.10 | 0.013 |
| 92 | Glutathione, oxidized | ESI+ | 479.93 | 613.1564 | 1.80 | 1.31 | 0.070 |
| 93 | 2-Hydroxyphenethylamine | ESI+ | 249.56 | 120.0805 | 1.53 | 6.24 | 0.010 |
| 94 | Norspermidine | ESI+ | 260.91 | 132.1371 | 3.49 | 3.18 | 0.007 |
| 95 | Triethanolamine | ESI+ | 153.70 | 150.1112 | 0.11 | 2.00 | 0.062 |
| 96 | Sphinganine (d17:0) | ESI+ | 35.69 | 288.2886 | 1.28 | 3.65 | 0.008 |
| 97 | Pro-Trp | ESI+ | 57.49 | 302.3040 | 1.13 | 1.96 | 0.097 |
| 98 | Arachidonoyl Thio-PC | ESI+ | 134.34 | 784.5831 | 0.88 | 7.28 | 0.004 |
| 99 | Methylpicolinate | ESI+ | 211.78 | 138.0541 | 0.57 | 2.76 | 0.001 |
| 100 | Serotonin | ESI+ | 227.99 | 160.0752 | 0.38 | 4.36 | 0.011 |
| 101 | 5-methyl-5-phenylhydantoin | ESI+ | 242.93 | 191.0829 | 0.57 | 1.52 | 0.004 |
| 102 | Torin 2 | ESI+ | 439.59 | 217.0806 | 1.22 | 2.31 | 0.025 |
| 103 | Carfentrazone-ethyl | ESI+ | 294.65 | 429.0551 | 0.74 | 1.19 | 0.002 |
| 104 | Bilirubin | ESI+ | 218.80 | 585.2685 | 0.63 | 1.43 | 0.016 |
| 105 | Daidzein | ESI+ | 24.39 | 255.0639 | 0.58 | 1.24 | 0.063 |
| 106 | Dihydro-alpha-ionone | ESI+ | 30.43 | 258.1853 | 4.04 | 1.04 | 0.065 |
| 107 | Geldanamycin | ESI+ | 196.41 | 583.2522 | 0.39 | 2.26 | 0.007 |
| 108 | Caylin-1 | ESI+ | 400.52 | 651.1076 | 1.13 | 1.23 | 0.108 |
