# Supplementary figures and images for: Plasma-based Raman spectroscopy for early detection of acute myocardial infarction in murine models
Source: Sci Rep. 2025 Dec 10;16:705. doi: 10.1038/s41598-025-30292-y (PMC12780145; doi:10.1038/s41598-025-30292-y)

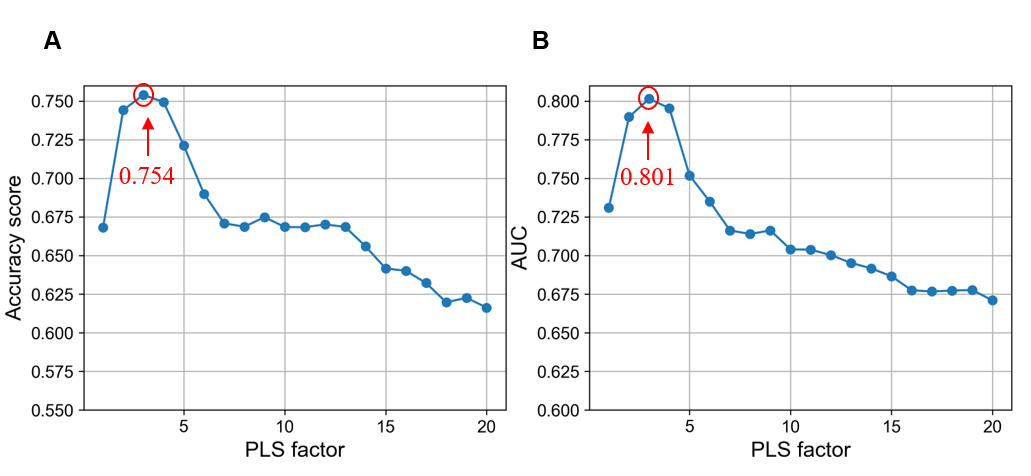

Supplement: Supplementary file 2 — Supplementary Material 2 [file 41598_2025_30292_MOESM2_ESM.tif]

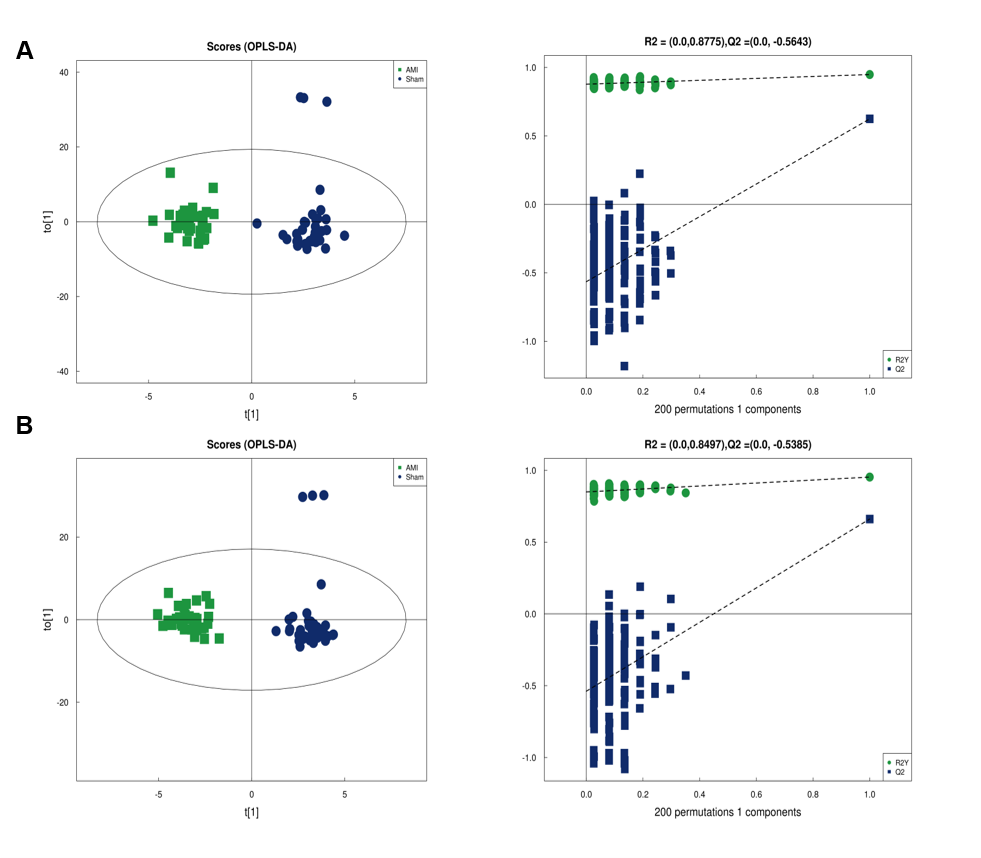

Supplement: Supplementary file 3 — Supplementary Material 3 [file 41598_2025_30292_MOESM3_ESM.tif]
